# Adaptive memory: The effects of survival-constrained retrieval on recognition depend on initial encoding conditions

**DOI:** 10.3758/s13421-025-01767-0

**Published:** 2025-08-11

**Authors:** Raoul Bell, Laura Mieth, Meike Kroneisen

**Affiliations:** 1 Department of Experimental Psychology, Heinrich Heine University Düsseldorf, 40225 Düsseldorf, Germany; 2 Department of Psychology, University of Kaiserslautern-Landau (RPTU), 76829 Landau, Germany

**Keywords:** Testing effect, Retrieval practice, Encoding-constrained retrieval, Retrieval-enhanced learning, Survival processing

## Abstract

Information relevant to survival has been found to be prioritized in memory, a finding often interpreted as reflecting evolved mnemonic mechanisms. While much research has focused on survival processing at encoding, the effect of constraining retrieval to the survival condition on later memory performance remains less well studied. Two experiments serve to examine whether survival-constrained retrieval in an intermediate source-constrained retrieval test impairs or improves recognition of words in a final memory test, depending on whether intermediate retrieval was constrained to the condition in which the words were initially encoded. Two competing hypotheses are evaluated: The retrieval-based interference account predicts that survival-constrained retrieval may blur the distinction between words initially judged for survival relevance and foils introduced in the intermediate source-constrained retrieval test, leading to impaired final recognition. In contrast, the retrieval-based strengthening account suggests that survival-constrained retrieval strengthens memory for the words initially judged for survival relevance, relative to words encoded in a control condition. Across two experiments using survival, pleasantness, or moving relevance judgments at initial encoding and intermediate source-constrained retrieval, final recognition was consistently better when intermediate retrieval was constrained to the initial encoding condition. The results contribute to the adaptive-memory framework by showing that survival-related memory advantages are shaped by both initial encoding and intermediate retrieval processes.

## Introduction

Properties of biological systems, including cognitive functions, are the product of evolution. Memory is no exception: the capacity to remember is a useful adaptation, shaped over evolutionary time to enhance the survival and the reproductive success of biological organisms. The central tenet of the adaptive-memory framework (Nairne, 2022; Nairne & Pandeirada, 2008, 2016) is that memory systems bear the imprint of these ancestral selection pressures. Therefore, information that was most relevant to our ancestors’ survival and reproductive needs should be more effectively encoded, stored, and retrieved. The adaptive nature of memory is evident in phenomena such as the heightened recall of information related to survival (Abel & Bäuml, 2013; Bonin et al., 2020; Nairne et al., 2007, 2008, 2019; Otgaar et al., 2010; Raymaekers et al., 2014; Stillman et al., 2014), child care (Bonin et al., 2020; Seitz et al., 2018), and social interactions (Bell & Buchner, 2012; Buchner et al., 2009; Çabuk-Çolak et al., in press); Kroneisen et al., 2021; Schaper et al., 2022; Schaper et al., 2019). The value of a theory lies in its ability to make accurate predictions, and the adaptive-memory framework has been productive in helping to discover new memory phenomena, including the animacy effect (Bonin et al., 2015, 2022; Félix et al., 2019; Komar et al., 2023, 2024; Meinhardt et al., 2018; Nairne et al., 2013, 2017) and the contamination effect (Bonin et al., 2019; Fernandes et al., 2017; Thiebaut et al., 2021).

Among the most robust memory phenomena discovered under the umbrella of the adaptive-memory framework is the *survival processing effect* (Nairne & Pandeirada, 2016; Nairne et al., 2007), referring to the observation that judging information for its survival relevance leads to enhanced memory. In a typical experiment, participants are asked to imagine being stranded in the grasslands of a foreign land without the basic means of survival, having to secure food and water and protect themselves against predators. They are presented with a list of words and asked to judge the relevance of each word for survival. In a control condition, participants are often asked to judge the pleasantness of the words, serving as a form of deep encoding, or to imagine moving to a foreign land, thereby controlling for the schematic nature of the processing task. After the incidental encoding phase, participants are given a surprise memory test. This test can take various forms, including free recall (Nairne & Pandeirada, 2010; Nairne et al., 2007, 2008), recognition (Nairne et al., 2007), and source memory (Kroneisen & Bell, 2018; Misirlisoy et al., 2019; Nairne et al., 2012). These experiments typically demonstrate that judging words for survival relevance leads to better memory than control conditions involving deep encoding processes (Nairne et al., 2008; Scofield et al., 2018). Theories on the survival processing effect have mostly focused on encoding processes (Burns et al., 2011; Howe & Otgaar, 2013; Klein et al., 2011; Otgaar et al., 2015; Smeets et al., 2012). The richness-of-encoding account (Bell et al., 2015b; Kroneisen & Erdfelder, 2011; Kroneisen et al., 2014, 2016; Röer et al., 2013) posits that survival processing leads to rich encoding, thereby enhancing free recall. Furthermore, survival processing may promote a combination of item-specific and relational encoding that is particularly effective for free recall (Burns et al., 2011, 2013; Howe & Otgaar, 2013). More recently, research has begun to examine how retrieval demands may contribute to the survival processing advantage. Specifically, two studies have shown that the survival processing effect can depend on the match between encoding operations and retrieval demands (Alban & Annibal, 2022; Dewhurst et al., 2023), consistent with the transfer-appropriate processing principle (Blaxton, 1989; Morris et al., 1977).

An interesting direction in the survival processing literature focuses on how constraining retrieval to the survival condition influences later memory. To investigate this, Nairne et al. (2015) used the established memory-for-foils paradigm (Jacoby et al., 2005). Nairne et al.’s (2015) experiments each consisted of three phases. In an initial incidental-encoding phase, participants judged words for their relevance to a survival scenario or a control condition (pleasantness or moving). In an intermediate source-constrained retrieval test, participants were asked whether each word had been initially judged for survival relevance (“Did you rate this word for survival?”) or for the control dimension (e.g., “Did you rate this word for pleasantness?”). In a final free recall test, participants were instructed to recall all words presented during the experiment: both the words that were initially judged and the foils that were newly introduced in the intermediate source-constrained retrieval test. Across all three experiments, Nairne et al. (2015) found that intermediate survival-constrained retrieval enhanced final recall of foils. These results are consistent with prior work demonstrating that constraining retrieval to deep encoding conditions improves subsequent memory for foils compared to constraining retrieval to shallow encoding conditions (Jacoby et al., 2005; Marsh et al., 2009), consistent with the view that survival processing represents a particularly deep form of encoding.

An influential account of the memory-for-foils effect is that source-constrained retrieval selectively reinstates the cognitive processes engaged during encoding (Jacoby et al., 2005; Vogelsang et al., 2016). When retrieval is constrained by a specific query, such as determining whether a word was initially judged for its relevance in a particular scenario, participants may engage in retrieval operations that resemble those used during initial encoding. This selective reinstatement of encoding operations may help generate retrieval cues that are specifically tailored to the query. In the survival-constrained retrieval condition, participants may reinstate the same rich processing that underlies the original survival memory advantage. For instance, when the source-constrained retrieval query prompts a participant to think about whether the word “spoon” had been judged for survival relevance during initial encoding, a participant may imagine the utility of the spoon in defending against predators, digging for water, or preparing food. Crucially, survival processing may be applied not only to words from the initial encoding phase but also to foils encountered for the first time during intermediate retrieval. The familiarity of the imagined uses or the fluency with which they are generated may then serve as cues for judging whether the word “spoon” had been initially judged for survival relevance. In the paradigm used by Nairne et al. (2015), which closely followed Jacoby et al.’s (2005) original memory-for-foils paradigm, survival-constrained retrieval was expected to enhance memory for foils. This hypothesis was tested in the final recall test, which required participants to recall all words encountered during the experiment, regardless of whether they had been initially encoded or newly introduced during intermediate retrieval. Intermediate survival-constrained retrieval may have enhanced elaboration of the foils, which in turn may have supported their retrieval in the final recall test.

However, from a mechanistic perspective, the same processing operations may be maladaptive when they do not align with the demands of the final memory test (cf. Zawadzka et al., 2017). Specifically, when a final recognition test requires participants to discriminate between words judged during initial encoding and foils introduced during intermediate retrieval, survival processing in both phases may blur this distinction. According to the *retrieval-based interference account*, overlap in processing between initial encoding and intermediate retrieval may reduce participants’ ability to discriminate words judged during initial encoding from foils, thereby decreasing final recognition. The reduction in recognition should be especially pronounced when initial survival processing is followed by survival-constrained retrieval, where the similarity in processing is assumed to be amplified by the elaborative nature of survival processing, relative to control conditions. To test this hypothesis, we used a final recognition test in Experiment 1 in which participants were instructed to accept only the words presented during initial encoding and to reject the foils introduced during intermediate retrieval. To examine source-specific confusion more directly, source memory was assessed in addition to recognition in Experiment 2. In the final source memory test, participants were asked to assign each recognized word to either the survival or the control (e.g., pleasantness) encoding condition, provided they believed the word had been processed during initial encoding. The retrieval-based interference account predicts that intermediate survival-constrained retrieval may lead to source confusion. This should result in reduced source memory in the survival condition relative to the control condition and an increased bias toward guessing survival as the source. In this way, the retrieval-based interference account challenges the broader assumption that survival processing consistently produces memory advantages over control conditions.

However, there is an alternative account that stands in direct contrast to the retrieval-based interference account. Source-constrained retrieval prompts selective retrieval of words processed in a specific condition. According to the retrieval-based strengthening account, final recognition should be enhanced, rather than decreased, for words that had to be retrieved, relative to words that did not have to be retrieved. This account builds upon the well-documented finding that having to retrieve specific information from memory can enhance subsequent memory for that same information (cf. Carpenter, 2009; McDaniel, 2023; Pierce et al., 2017; Roediger & Karpicke, 2006). Critically, such retrieval-induced strengthening of memory occurs specifically for information that had to be retrieved, or for closely associated information, but typically not for unrelated encoded information that did not have to be retrieved (cf. Pan & Rickard, 2018). From the perspective of the adaptive-memory framework, one might further predict that retrieval-based strengthening should be particularly pronounced when words had not only been judged for survival relevance but also subsequently had to be retrieved in the survival condition because information that had to be both encoded and retrieved within a survival context is especially important to remember. Thus, intermediate survival-constrained retrieval could yield especially robust recognition benefits for words initially judged for survival relevance relative to the words processed in the control condition.

Two experiments tested these competing predictions using a source-constrained retrieval test followed by either a recognition test (Experiment 1) or a source memory test (Experiment 2). Given the parallel structure of the hypothesis tests across both experiments, we first report the methods and results of Experiments 1 and 2 before jointly discussing the findings in the *General discussion*.

## Experiment 1

### Methods

#### Participants

The online experiment was implemented using SoSci Survey (Leiner, 2019) and made available through https://www.soscisurvey.de. All participants confirmed that they were ≥ 18 years of age and reported having adequate visual abilities. Participation was only possible with a desktop or laptop computer. Before starting the study, participants were asked to participate alone in a distraction-free environment. The experiment was advertised on social media and via email. The data of nine participants who started the incidental learning phase had to be excluded because these participants did not complete the experiment or withdrew their consent into the use of their data. The final data set comprised the data of *N* = 97 participants (87 female, nine male, one non-binary) with a mean age of 23 (*SD* = 6) years. All participants had at least a university entrance qualification. A sensitivity analysis with G*Power (Faul et al., 2007) showed that, with a sample size of *N* = 97 participants and 160 decisions in the recognition test, a survival processing effect on recognition (*df* = 1) as small as *w* = 0.03 could be detected at an α level of 0.05 with a statistical power of 1 − β = 0.95.

#### Materials

We used the same set of 160 German words previously used by Kroneisen and Bell (2018). These words were drawn from ten different categories (animals, clothes, fruits, furniture, musical instruments, professions, sports, tools, vegetables, and vehicles), originally compiled in the database of Schröder et al. (2012). English translations of all words from the list are available in the online supplementary material of Kroneisen and Bell (2018).

#### Procedure

The experiment consisted of an incidental learning phase and two consecutive memory tests: an intermediate source-constrained retrieval test and a final recognition test.

##### Incidental learning phase

 Participants first received the instructions on the judgment task:

In the first phase of the experiment you have a judgment task. You will see a list of words and will be asked to judge each word in one of two ways. For some words, you will be asked to judge the relevance of the words to survival in a situation as described below. For other words, you will have to judge their pleasantness.

For the survival situation, please imagine that you are stranded in the grasslands of a foreign land without the basic means of survival. Over the next few months, you will need to find steady supplies of food and water and protect yourself from predators. Please rate how relevant each word will be for you to survive in this situation. The relevance rating scale ranges from 1 to 5, with 1 being totally irrelevant and 5 being extremely relevant. Some words may be relevant, others may not – it is up to you to judge.

In other cases, you are asked to judge the pleasantness of the word in question. The pleasantness rating scale ranges from 1 to 5, with 1 being totally unpleasant and 5 being extremely pleasant. Some words may be pleasant, others not – it is up to you to judge.

For each participant, a different subset of 80 words were randomly selected from the pool of 160 words to be presented in the incidental learning phase. The words in the study were randomly assigned to two conditions. Half were judged for survival relevance and the other half for pleasantness. To facilitate participants’ immersion in the scenarios, the incidental learning phase was divided into four blocks. In each block, participants judged the words either for survival relevance or pleasantness, in an alternating fashion. For example, a participant may judge the first 20 words for survival relevance, the next 20 for pleasantness, followed by another 20 for survival relevance and the final 20 again for pleasantness. The starting point of the incidental learning phase – whether with survival relevance or pleasantness judgments – was counterbalanced among participants. The words were randomly assigned to the blocks and presented in a random order within these blocks. The words were presented one at a time at the center of the browser window. Above each word, participants were either asked “Please judge this word for its relevance for survival” or “Please judge this word for its pleasantness.” To facilitate the discrimination between the two judgment tasks, the word “survival” was always displayed in red font and “pleasantness” in blue font. Participants used a 5-point scale to rate either the survival relevance (totally irrelevant, rather irrelevant, neither nor, rather relevant, extremely relevant) or pleasantness (totally unpleasant, rather unpleasant, neither nor, rather pleasant, extremely pleasant) of the words. The ratings were somewhat lower for survival relevance (*M* = 2.62, *SD* = 0.44) than for pleasantness (*M* = 3.08, *SD* = 0.26).

##### First distractor task 

When the judgment phase was completed, a first distractor task followed in which participants were asked to solve ten simple math problems such as “17 − 9” by typing the solutions into text fields. On average, participants solved 9.67 (*SD* = 0.75) of the ten problems correctly. The median duration of the distractor task was 39 s.

##### Intermediate source-constrained retrieval test

Participants then received the instructions for the source-constrained retrieval test:

In the following memory test, you will be presented with a series of words. For each word, please remember whether you have judged the word for its relevance to survival or its pleasantness in the first phase of the experiment. You answer by selecting yes or no.

Participants were shown the 80 words they had initially judged, along with 80 foils. Half of the words from each judgment type (survival relevance, pleasantness) and half of the foils were presented with a query constraining retrieval to words initially judged for survival relevance; the other half were presented with a query constraining retrieval to words initially judged for pleasantness. To immerse the participants in the retrieval task, the source-constrained retrieval test was divided into four blocks. Retrieval was constrained to either the survival or the pleasantness condition, with the query alternating across blocks. For example, retrieval could be constrained to survival for the first 40 words, then to pleasantness for the next 40 words, followed by survival-constrained retrieval for another set of 40 words and pleasantness-constrained retrieval for the final 40 words. It was counterbalanced across participants whether the source-constrained retrieval test started with survival-constrained or pleasantness-constrained retrieval. The words were randomly assigned to the blocks and presented with a random order within these blocks. The words were presented one at a time at the center of the browser window. Above each word, participants were asked either “Did you judge the word’s relevance to survival in the first phase of the experiment?” or “Did you judge the word’s pleasantness in the first phase of the experiment?” To help participants to differentiate between the two queries, the word “survival” was always displayed in red font and the word “pleasantness” in blue font. Participants responded to each query with either “yes” or “no.” Words initially judged for survival relevance received more “yes” answers in response to the query constraining retrieval to the survival condition (*M* = 0.84, *SD* = 0.12) than words initially judged for pleasantness (*M* = 0.27, *SD* = 0.20) or foils (*M* = 0.06, *SD* = 0.10). Similarly, words initially judged for pleasantness received more “yes” answers in response to the query constraining retrieval to the pleasantness condition (*M* = 0.80, *SD* = 0.16) than words initially judged for survival relevance (*M* = 0.19, *SD* = 0.19) or foils (*M* = 0.05, *SD* = 0.10). However, due to the limited data categories in the source-constrained retrieval test, it is impossible to separate recognition, source memory, and guessing. These results are therefore not analyzed further, as the main purpose of the source-constrained retrieval test was to constrain retrieval to examine the mnemonic effects of this manipulation in the final recognition test, not to measure these processes. For those interested in recognition, source memory, and guessing, we conducted a detailed analysis in the final source memory test of Experiment 2 (see *Results* section of Experiment 2).

##### Second distractor task 

When the source-constrained retrieval test was completed, a second distractor task followed. On average, participants solved 9.78 (*SD* = 0.46) of the ten math problems correctly. The median duration of the distractor task was 38 s.

##### Final recognition test

 Participants then received the instructions for the final recognition test:

In the following memory test you will be presented with a series of words again. For each word, please remember whether you have judged the word in the first phase of the experiment or not. If you have judged the word in the first phase of the experiment, then answer yes. If you have not judged the word in the first phase of the experiment, then answer no.

Participants were then shown all of the 160 words in an individually determined, random order. The words were presented one at a time at the center of the browser window. Above each word, the participants were asked “Have you judged the word in the first phase of the experiment?” The participants responded to the question with either “yes” or “no.”

### Results

In Table 1, we report the hit rates and false alarm rates in the final recognition test that formed the basis of the model-based analysis reported below.

**Table 1:** Proportions of “yes” and “no” responses to the question “Have you judged the word in the first phase of the experiment?” in the recognition test of Experiment 1 as a function of word type (words judged for survival relevance, words judged for pleasantness, foils) and retrieval condition (survival-constrained retrieval, pleasantness-constrained retrieval)

	Survival-constrained retrieval		Pleasantness-constrained retrieval	
	“Yes”	“No”	“Yes”	“No”
Words judged for survival relevance	0.85	0.15	0.81	0.19
Words judged for pleasantness	0.81	0.19	0.84	0.16
Foils	0.17	0.83	0.17	0.83

**Fig. 1:**
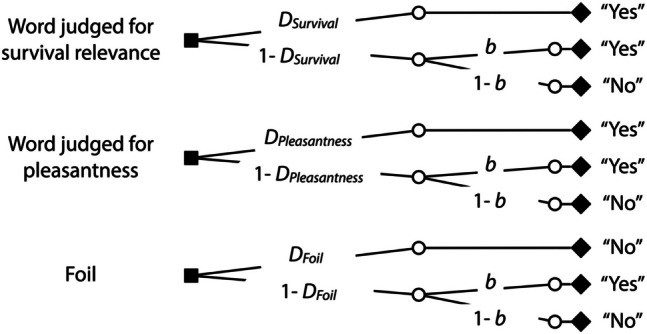
The two-high threshold word-recognition model illustrated in the form of processing trees. For each type of words included in the recognition test (words judged for survival relevance, words judged for pleasantness, foils), a separate processing tree is displayed. The trees lead to the responses that can be observed in the recognition test (“yes” or “no” in response to the question “Have you judged the word in the first phase of the experiment?”). The letters along the branches represent the probabilities of the postulated latent processes (*D*: recognition, *b*: guessing “old”)

Multinomial processing tree models serve to estimate probabilities of latent cognitive states from observable responses. In memory research, these models are used to disentangle memory and guessing processes (Bröder & Meiser, 2007; Erdfelder et al., 2007; Meiser & Bröder, 2002; Rummel et al., 2011; Smith & Bayen, 2004). The two-high threshold word-recognition model is one of the simplest models due to its straightforward structure. Here we use the two-high threshold word-recognition model that has been favorably evaluated in validation studies (Bayen et al., 1996; Snodgrass & Corwin, 1988). The model is illustrated in Fig. 1. This model has three trees, each for a category of words in the recognition test: words initially judged for relevance to survival in the grasslands, words initially judged for pleasantness, and foils that had not been judged in the incidental learning phase. To illustrate, a word that had been initially judged for survival relevance is recognized as having been present at encoding with probability *D*_Survival_. If the participant fails to recognize the word with probability 1 − *D*_Survival_, the participant may still guess that the word has been initially judged with probability *b*. With probability 1 − *b*, the word is falsely rejected. Parallel processes are assumed to occur in the trees referring to words that had been initially judged for pleasantness and foils that had not been judged. For instance, the participant may recognize a foil as not having been present at encoding with probability *D*_Foil_, in which case the foil is correctly rejected. If the participant fails to recognize the foil with probability 1 − *D*_Foil_, guessing is used in the same way as in response to unrecognized words.

**Fig. 2:**
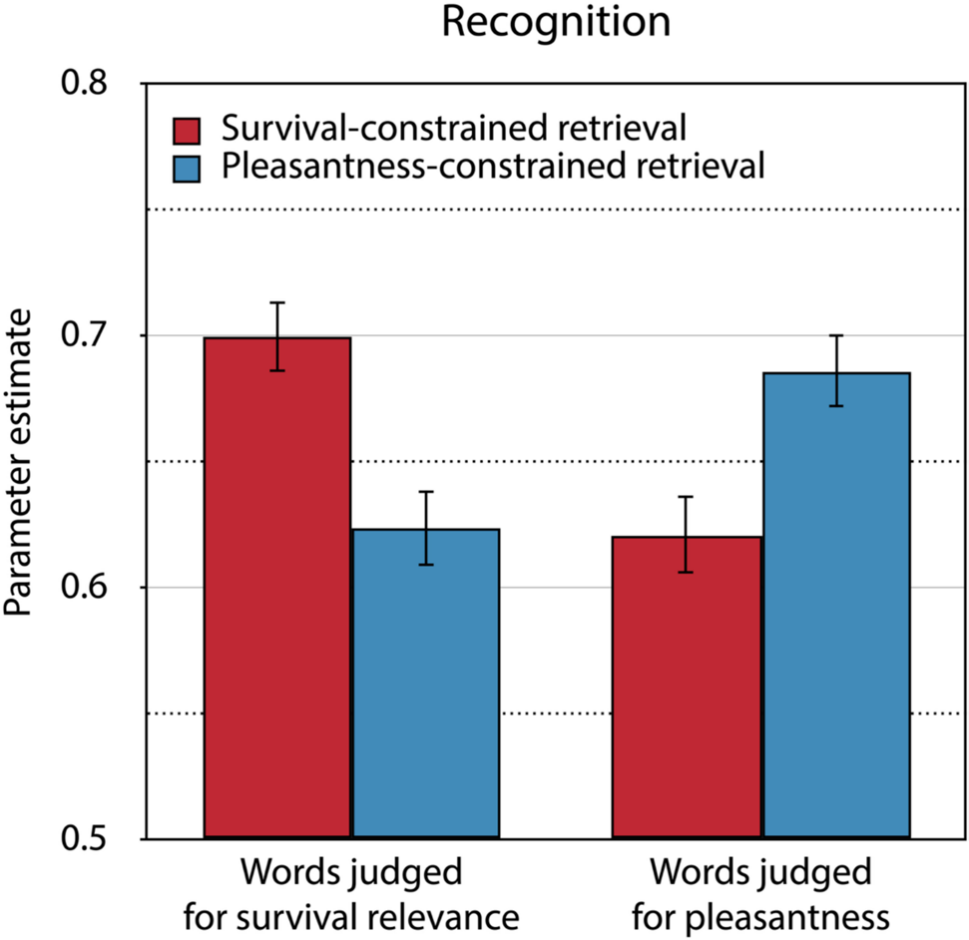
Estimates of parameter *D* reflecting recognition in the final memory test of Experiment 1. Recognition for words judged for survival and pleasantness at initial encoding is presented as a function of whether retrieval had been constrained to the survival or the pleasantness condition in the source-constrained retrieval test. The error bars represent the standard errors

For the model-based analysis, the α level was set to 0.05. Parameter estimates and goodness-of-fit tests were obtained using multiTree (Moshagen, 2010). Two sets of the trees of the recognition model depicted in Fig. 1 are needed, one for the words for which retrieval had been constrained to the survival condition and one for the words for which retrieval had been constrained to the pleasantness condition in the source-constrained retrieval test. When using a multinomial processing tree model to test hypotheses, it is important to first establish a base model that fits the data adequately. This involves ensuring that the structure of the base model is consistent with the observed data. The model fit is evaluated by assessing the discrepancy between the data that are observed and the data that are predicted by the model, using the goodness-of-fit statistic *G*^2^, which is chi-square distributed with degrees of freedom specified in parentheses. A satisfactory model fit is indicated by a non-significant *G*^2^ value above the significance threshold of 0.05. To obtain an identifiable base model, we assumed that *D*_Foil_ = (*D*_Survival_ + *D*_Pleasantness_) ÷ 2, following the procedure described in Bell et al., (2015a). The resulting model is a saturated model which perfectly fits the data. To be able to achieve a quantifiable model fit, we also incorporated the assumption that the bias to guess “old,” represented by parameter *b*, does not differ as a function of the retrieval condition into the base model. This model adequately reflects the data, *G*^2^(1) = 0.03, *p* = 0.865. Parameter *b* was estimated to be 0.50 (*SE* = 0.01). Figure 2 displays the estimates of the recognition parameter separately for words initially judged for survival relevance and words initially judged for pleasantness as a function of whether retrieval was constrained to the survival or the pleasantness condition. Supplementary ANOVA-based analyses of the recognition results of both experiments are available at the Open Science Framework (OSF) project page (see “Availability of data and materials” section).

Multinomial processing tree models allow hypotheses to be tested directly at the level of the parameters representing the latent cognitive processes assumed to underlie the observable behavior. The hypothesis that recognition is enhanced for words that had been initially judged for survival relevance in comparison to words that had been initially judged for pleasantness can be tested by restricting the recognition parameters to be equal across these conditions. If this equality restriction leads to a statistically significant increase in the Δ*G*^2^ statistic, reflecting the difference in fit between the nested model with the equality restriction compared to the base model without this restriction, then it can be concluded that recognition differed significantly between the two conditions. As is already obvious from the estimates of the recognition parameters displayed in Fig. 2, the typical survival processing advantage in recognition was only obtained for words for which retrieval had been constrained to the survival condition in the source-constrained retrieval test, Δ*G*^2^(1) = 10.59, *p* = 0.001, while it was significantly reversed for those words for which retrieval had been constrained to the pleasantness condition in the source-constrained retrieval test, Δ*G*^2^(1) = 6.70, *p* = 0.010. Furthermore, constraining retrieval to the survival condition led only to a recognition advantage for those words that had been initially judged for survival relevance, Δ*G*^2^(1) = 13.34, *p* < 0.001, while constraining retrieval to the pleasantness condition led to enhanced recognition for those words that had been initially judged for pleasantness, Δ*G*^2^(1) = 9.55, *p* = 0.002.

## Experiment 2

Experiment 2 extended the test of the competing accounts in two key ways. First, we replaced the pleasantness control condition with a new control condition involving judgments about the relevance of words for moving to a foreign land. This allowed us to assess the robustness of the recognition effects using different control conditions, following the research strategy of Nairne et al. (2015). Second, we assessed source memory in addition to recognition in the final memory test by asking participants to attribute recognized words to their initial encoding condition. While the results of Experiment 1 did not support the retrieval-based interference account, Experiment 2 tests an additional prediction of this account concerning source memory which was not examined in Experiment 1: If survival-constrained retrieval mirrors survival processing at initial encoding, it may lead to source confusion, reducing source memory for words initially processed for survival and inducing a bias toward guessing survival as the source.

### Methods

#### Participants

The online experiment was implemented using SoSci Survey (Leiner, 2019) and made available through https://www.soscisurvey.de. All participants reported having adequate visual abilities. Participation was only possible with a desktop or laptop computer. Before starting the study, participants were asked to participate alone in a distraction-free environment. The experiment was advertised on social media and via email. The data of 18 participants who started the incidental learning phase had to be excluded because these participants did not complete the experiment or withdrew their consent into the use of their data. One data set had to be excluded because the participant was < 18 years of age and thus legally could not consent into the use of their data. The final data set comprised the data of *N* = 98 participants (84 female, 14 male) with a mean age of 22 (*SD* = 5) years. All participants but one had at least a university entrance qualification. A sensitivity analysis with G*Power (Faul et al., 2007) showed that, with a sample size of *N* = 98 participants and 160 decisions in the source-memory test, a survival processing effect on recognition (*df* = 1) as small as *w* = 0.03 could be detected at an α level of 0.05 with a statistical power of 1 – β = 0.95.

#### Materials and procedure

The experiment consisted of an incidental learning phase and two consecutive memory tests: an intermediate source-constrained retrieval test and a final source memory test.

##### Incidental learning phase 

The incidental learning phase was parallel to Experiment 1, but participants now judged word relevance for a moving scenario instead of pleasantness. The instructions for the moving scenario read:

For the moving situation, please imagine that you are planning to move to a new home in a foreign country. In the next few months, you'll need to buy a new house and find help moving your belongings. Please judge how relevant to moving each word would be for you in this situation.

Above each word, participants were either asked “How relevant is the word for survival?” or “How relevant is the word to moving?” The ratings were somewhat higher for the judgment of survival relevance (*M* = 2.64, *SD* = 0.47) than for the judgment of moving relevance (*M* = 2.19, *SD* = 0.47).

##### First distractor task

 On average, participants solved 9.70 (*SD* = 0.58) of the ten problems correctly. The median duration of the distractor task was 40 s.

##### Intermediate source-constrained retrieval test

The source-constrained retrieval test was identical to that implemented in Experiment 1 with the only difference that participants were asked to retrieve whether they judged the word’s relevance to moving to a foreign country instead of the word’s pleasantness in the control condition of the first phase of the experiment. Words that had been judged for survival relevance received more “yes” answers in response to the query constraining retrieval to the survival condition (*M* = 0.82, *SD* = 0.14) than words that had been judged for moving relevance (*M* = 0.37, *SD* = 0.19) or foils (*M* = 0.07, *SD* = 0.08). Furthermore, words that had been judged for moving relevance received more “yes” answers in response to the query constraining retrieval to the moving condition (*M* = 0.74, *SD* = 0.16) than words that had been judged for survival relevance (*M* = 0.28, *SD* = 0.19) or foils (*M* = 0.08, *SD* = 0.10).

##### Second distractor task

On average, participants solved 9.77 (*SD* = 0.57) of the ten problems correctly. The median duration of the distractor task was 39 s.

##### Final source memory test

 Participants then received the instructions for the final source memory test:

In the following memory test you will be presented with a series of words again. For each word, please remember whether you have judged the word in the first phase of the experiment or not. If you have judged the word in the first phase of the experiment, then answer yes. If you have not judged the word in the first phase of the experiment, then answer no. If you answer yes, then you should also indicate whether you assessed the word's relevance to survival or moving in the first phase of the experiment.

Participants were then shown all of the 160 words in an individually determined, random order. The words were presented one at a time at the center of the browser window. Above each word, the participants were asked “Have you judged the word in the first phase of the experiment?” The participants responded to the question with either “yes” or “no”. If participants answered with “yes”, they were also asked “Have you judged the word for its relevance to survival or moving in the first phase of the experiment?” The participants responded to the question with “survival” or “moving”.

### Results

In Table 2, we report the proportion of answers in the final source-memory test that formed the basis of the model-based analysis reported below.

**Table 2:** Proportions of “yes” and “no” responses to the question “Have you judged the word in the first phase of the experiment?” and “survival” versus “moving” answers in the source memory test of Experiment 2 as a function of word type (words judged for survival relevance, words judged for moving relevance, foils) and retrieval condition (survival-constrained retrieval, moving-constrained retrieval)

	Survival-constrained retrieval			Moving-constrained retrieval		
	“Yes”		“No”	“Yes”		“No”
	“Survival”	“Moving”		“Survival”	“Moving”	
Words judged for survival relevance	0.70	0.11	0.19	0.61	0.17	0.22
Words judged for moving relevance	0.23	0.51	0.26	0.17	0.61	0.23
Foils	0.07	0.08	0.85	0.06	0.11	0.84

**Fig. 3:**
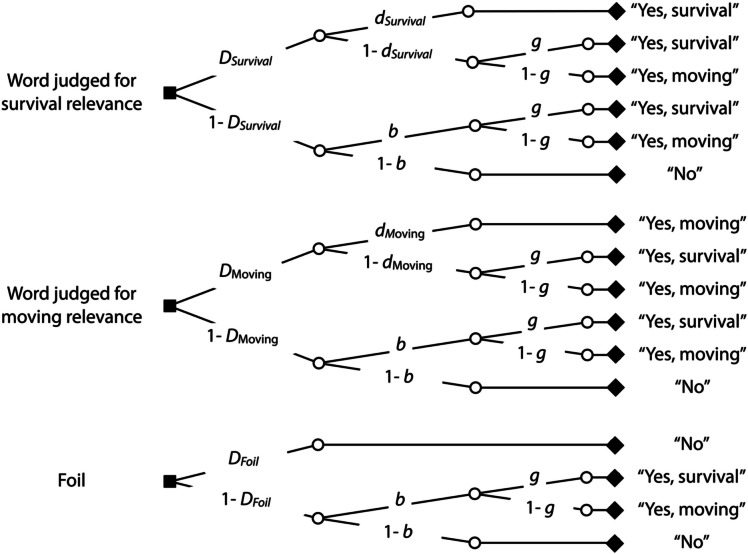
The two-high threshold source memory model illustrated in the form of processing trees. For each type of words included in the source memory test (words judged for survival relevance, words judged for moving relevance, foils), a separate processing tree is displayed. The trees lead to the responses that can be observed in the source memory test (“survival”, “moving,” or “no” in response to the question “Have you judged the word for its relevance to survival or moving in the first phase of the experiment?”). The letters along the branches represent the probabilities of the postulated latent processes (*D*: Recognition, *d*: source memory, *b*: guessing “old”, *g*: guessing “survival”)

The source memory model (Bayen et al., 1996) is one of the most widely used and best-validated multinomial processing tree models in cognitive psychology and has been extensively used to disentangle recognition, source memory, and guessing (for reviews, see Erdfelder et al., 2009; Kuhlmann et al., 2021). The model is illustrated in Fig. 3. This model has three trees, one for each type of words judged in the source memory test: words initially judged for their relevance to survival in the grasslands, words initially judged for their relevance to moving to a foreign country, and foils that had not been judged in the incidental learning phase. The source memory model is a straightforward extension of the recognition model displayed in Fig. 1. To illustrate, a word that had been initially judged for survival relevance is recognized as having been present at encoding with probability *D*_Survival_. The participant may also have source memory for the fact that the word has been initially judged for survival relevance with probability *d*_Survival_. If the participant fails to remember the specific context in which the word has been encountered with probability 1 − *d*_Survival_, the participant may still correctly guess that the word has been judged for survival relevance with probability *g*. With probability 1 − *g*, the participant may falsely guess that the word has been judged for moving relevance. If the participant fails to recognize the word as having been present at encoding with probability 1 − *D*_Survival_, the participant may still guess that the unrecognized word is old with probability *b*. Participants may then guess that the word has been judged for survival relevance with probability *g* or for moving relevance with probability 1 − *g*. With probability 1 − *b*, the word is falsely guessed to have not been present in the incidental learning phase. Parallel processes are assumed to occur in the trees referring to words that have been initially judged for moving relevance and foils. For instance, in case of foils, the participant may recognize the foil as not having been present at encoding with probability *D*_Foil_, in which case the word is correctly rejected. If the participant fails to recognize the foil with probability 1 – *D*_Foil_, the same guessing processes are used as in response to unrecognized old words.

**Fig. 4:**
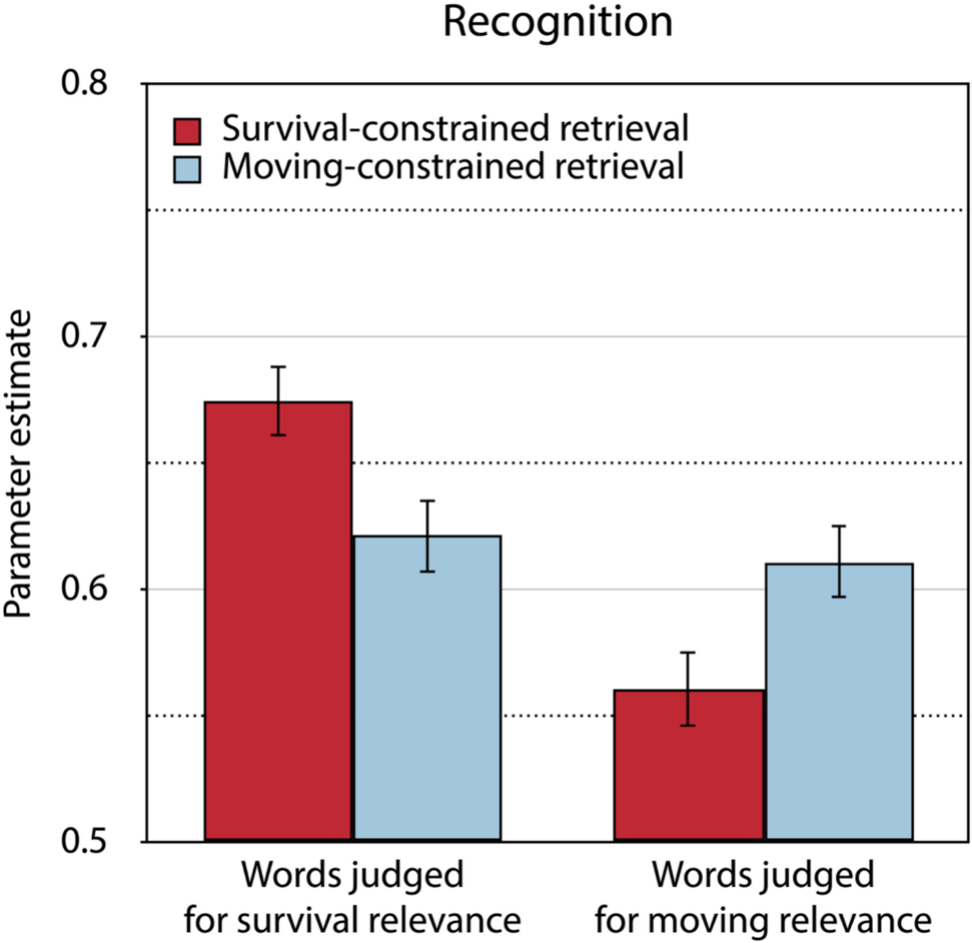
Estimates of parameter *D* reflecting recognition in the final memory test of Experiment 2. Recognition for words judged for survival and moving relevance at initial encoding is presented as a function of whether retrieval had been constrained to the survival condition or the moving condition in the source-constrained retrieval test. The error bars represent the standard errors

Two sets of the trees of the source memory model depicted in Fig. 3 are needed, one for the words for which retrieval had been constrained to the survival condition and one for the words for which retrieval had been constrained to the moving condition in the source-constrained retrieval test. Parallel to Experiment 1, we assumed that *D*_Foil_ = (*D*_Survival_ + *D*_Moving_) ÷ 2, following the procedure described in Bell et al., (2015a), to obtain an identifiable base model. The resulting model is a saturated model which perfectly fits the data. Parallel to Experiment 1, we incorporated the assumption that the bias to guess “old,” represented by parameter *b*, does not differ as a function of the retrieval condition into the base model to achieve a quantifiable model fit. This model adequately reflects the data, *G*^2^(1) = 0.92, *p* = 0.338. Parameter *b* was estimated to be 0.41 (*SE* = 0.01). Figure 4 displays the estimates of the recognition parameter separately for words initially judged for survival and moving relevance as a function of whether retrieval was constrained to the survival condition or the moving condition.

As shown in Fig. 4, the typical survival processing advantage in recognition was only obtained for words for which retrieval had been constrained to the survival condition in the source-constrained retrieval test, Δ*G*^2^(1) = 25.40, *p* < 0.001, while it was absent for those words for which retrieval had been constrained to the moving condition in the source-constrained retrieval test, Δ*G*^2^(1) = 0.21, *p* = 0.645. Furthermore, constraining retrieval to the survival condition led only to a recognition advantage for those words that had been initially judged for survival relevance, Δ*G*^2^(1) = 7.21, *p* = 0.007, while constraining retrieval to the moving condition led to enhanced recognition for those words that had been initially judged for moving relevance, Δ*G*^2^(1) = 5.75, *p* = 0.017.

**Fig. 5:**
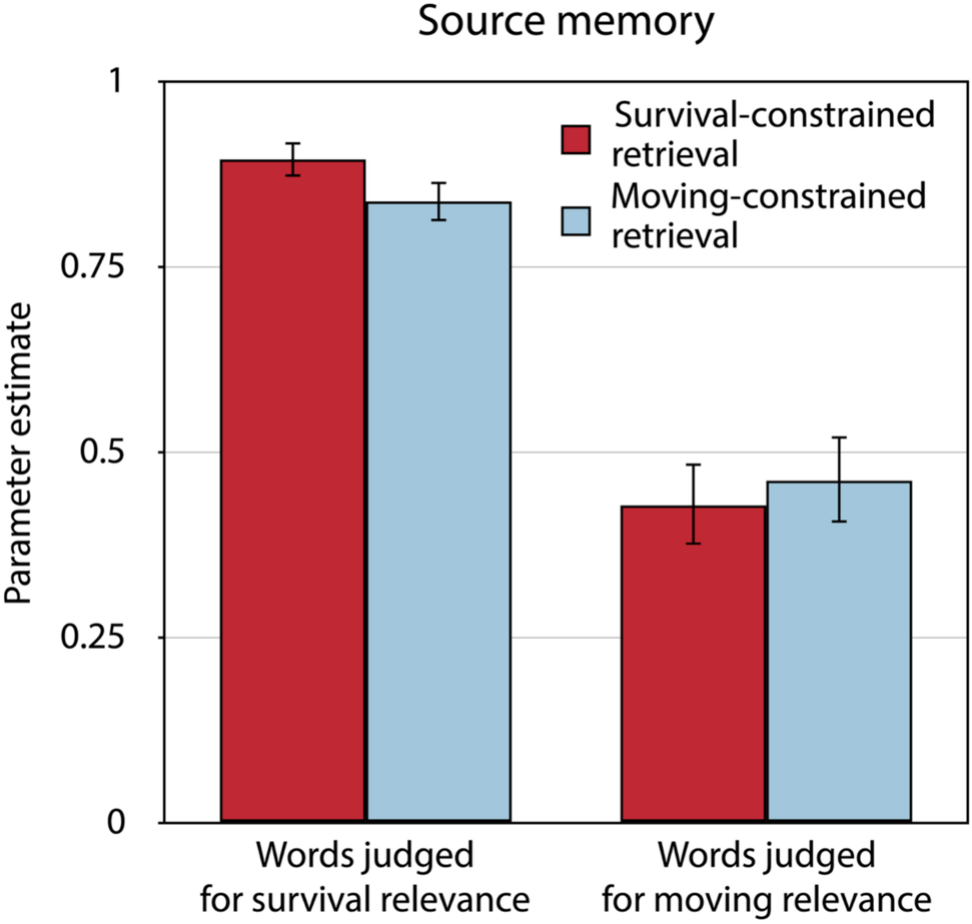
Estimates of parameter *d* reflecting source memory in the final memory test of Experiment 2. Source memory for words judged for survival and moving relevance at initial encoding is presented as a function of whether retrieval had been constrained to the survival condition or to the moving condition in the source-constrained retrieval test. The error bars represent the standard errors

Judging survival relevance at initial encoding led to better source memory than judging moving relevance, both for words for which retrieval had been constrained to the survival condition in the source-constrained retrieval test, Δ*G*^2^(1) = 57.95, *p* < 0.001, and for words for which retrieval had been constrained to the moving condition in the source-constrained retrieval test, Δ*G*^2^(1) = 33.75, *p* < 0.001 (Fig. 5). Furthermore, the retrieval condition had no significant effect on source memory, neither for those words that had been initially judged for survival relevance, Δ*G*^2^(1) = 2.56, *p* = 0.110, nor for those words that had been initially judged for moving relevance, Δ*G*^2^(1) = 0.17, *p* = 0.679. The source guessing parameter representing the probability of guessing that a word had been judged for survival rather than for moving relevance was estimated to be 0.45 (*SE* = 0.02) for words for which retrieval had been constrained to the survival condition and 0.35 (*SE* = 0.02) for words for which retrieval had been constrained to the moving condition in the source-constrained retrieval test. The fact that these estimates fall below 0.50 suggests that people showed a bias toward guessing that words were encoded in the moving condition regardless of retrieval conditions. However, this bias to guess that words had been judged for moving relevance was less pronounced when retrieval had been constrained to the survival condition than when retrieval had been constrained to the moving condition, Δ*G*^2^(1) = 15.04, *p* < 0.001.

## General discussion

The present experiments tested two competing accounts of how survival-constrained retrieval affects memory: the retrieval-based strengthening account and the retrieval-based interference account. While both accounts are grounded in the idea that survival processing involves rich and elaborate cognitive operations (Bell et al., 2015a; Kroneisen & Erdfelder, 2011; Kroneisen et al., 2014, 2016; Röer et al., 2013), they differ fundamentally in their predictions. The retrieval-based strengthening account predicts that intermediate survival-constrained retrieval strengthens memory for words encoded in the survival condition relative to words encoded in a control condition, leading to improved final recognition. In contrast, the retrieval-based interference account implies that engaging in survival processing at intermediate retrieval can blur the distinction between the words that were initially judged for survival relevance and the foils newly introduced at intermediate retrieval, thereby impairing final recognition and source memory. Across two experiments, we tested these competing hypotheses by assessing both recognition (Experiments 1 and 2) and source memory (Experiment 2). The results provide insights into how the combination of initial survival processing and intermediate survival-constrained retrieval determines final recognition and source memory.

The results on final recognition from both experiments are consistent with the predictions of the retrieval-based strengthening account and with the broader view that retrieval efforts effectively enhance subsequent memory specifically for information that had to be retrieved (Dudukovic et al., 2009; Hong et al., 2019; Roediger & Karpicke, 2006; Whiffen & Karpicke, 2017), relative to unrelated encoded information that did not have to be retrieved (Pan & Rickard, 2018). In line with this account, final recognition was numerically highest when words initially judged for survival relevance subsequently had to be retrieved, supporting the idea that survival-constrained retrieval strengthens, rather than reduces, memory for words encoded in the survival condition. More generally, final recognition benefited when intermediate retrieval was constrained to the same condition under which the words had been initially processed, irrespective of whether retrieval was constrained to the survival or a control condition. Thus, retrieval-based strengthening may reflect a general advantage of having to retrieve previously encoded words.

The results from both experiments provide little empirical support for the retrieval-based interference account. According to this account, engaging in survival processing at both initial encoding and intermediate retrieval should impair participants’ ability to discriminate words from the initial encoding phase from the foils due to overlapping processing across the two phases, thereby reducing final recognition. However, recognition was highest, not lowest, when initial survival processing was followed by survival-constrained retrieval, directly contradicting the account’s central prediction. Similarly, the source memory results showed that initial survival processing led to better source memory regardless of the intermediate retrieval condition, inconsistent with the prediction that survival-constrained retrieval would interfere with source memory for words initially encoded in a survival context. Although source guessing varied as a function of retrieval condition, participants consistently showed a bias toward guessing that words were encoded in the moving condition, with no clear evidence of a bias toward attributing words to the survival context following survival-constrained retrieval. Taken together, the retrieval-based interference account was not supported by the hypothesis test.

The present study builds on a previous study by Nairne et al. (2015), which followed the memory-for-foils paradigm introduced by Jacoby et al. (2005). Nairne et al. (2015) did not analyze whether survival-constrained retrieval benefits recall of words initially judged for survival relevance more than words initially judged in the control conditions because this question was tangential to their primary research aim. Nevertheless, the descriptive data reported in an appendix show a trend suggesting the survival processing advantage was more pronounced when the query constrained retrieval to the survival condition rather than to the control condition. Nairne et al.’s (2015) primary research aim was to test whether intermediate survival-constrained retrieval enhanced subsequent recall of foils in a free recall test, a hypothesis that was confirmed, consistent with prior research demonstrating that constraining retrieval to deep encoding conditions can improve subsequent memory for foils (Jacoby et al., 2005; Marsh et al., 2009). The present experiments were not designed to measure recall of foils but instead used a final recognition test in which participants were asked to accept only words from the initial encoding phase and reject foils. While neither of the present experiments provided evidence that survival-constrained retrieval increased the false acceptance of foils in the final recognition test, this does not imply a direct discrepancy between studies due to the differences in the test formats: Nairne et al.’s (2015) final free-recall test required participants to recall all previously encountered words, including the foils, whereas the present final recognition test required participants to accept only words from the initial encoding phase and to reject the foils.

In addition to the memory-for-foils effect following survival-constrained retrieval, Nairne et al. (2015) reported that initial survival processing had no effect on source memory, contrasting with the results of the final source memory test in the present Experiment 2. This discrepancy likely arises from the differences in how source memory was assessed. Nairne et al. (2015) measured source memory using the intermediate source-constrained retrieval task, in which participants responded “yes” or “no” to the source queries (e.g., “Did you rate this word for survival?”), without separately answering whether a word had initially been seen or not, and, if so, in which encoding condition. This limited data structure makes it difficult to clearly separate source memory from recognition or guessing biases. For example, for words initially judged for survival relevance, a “no” response to the survival query might reflect a recognition failure, a source memory failure, a bias toward guessing that the word was not presented or presented in a control condition. In contrast, the final source memory test in the present study yielded a richer data structure, requiring participants to first indicate whether each word had been initially seen or not, and, if so, in which condition. This allowed us to apply the well-validated multinomial source monitoring model (Bayen et al., 1996; Bröder & Meiser, 2007), to effectively disentangle recognition, source memory, and guessing processes. Using this approach, we found enhanced source memory for words initially judged for survival relevance, consistent with prior model-based analyses (Kroneisen & Bell, 2018; Misirlisoy et al., 2019).

A central assumption of the adaptive-memory framework (Nairne, 2022; Nairne & Pandeirada, 2008, 2016) is that human memory is tuned to prioritize information with relevance for survival, resulting in superior retention for information judged for survival relevance. The present experiments critically examined this assumption by testing predictions derived from the retrieval-based interference account. Specifically, this account implies that survival processing at both encoding and retrieval might impair recognition and source memory due to overlapping processing. These predictions were not supported by the present experiments. Instead, Experiment 2 showed that source memory was enhanced for words encoded in a survival context regardless of the retrieval condition. At the same time, the results also reveal boundary conditions for the survival memory advantage in recognition. Survival processing at encoding improved recognition relative to control conditions only when paired with survival-constrained retrieval. When retrieval was constrained to the control conditions, the survival processing memory advantage in recognition was either eliminated (Experiment 2) or reversed (Experiment 1). Likewise, survival-constrained retrieval enhanced final recognition only for words initially judged for survival relevance and decreased final recognition for words initially judged in the control conditions. This could be interpreted in terms of an adaptive mechanism because information that already had to be retrieved in the past, regardless of the type of context, may be more likely to be needed again in the future than information that only had to be encoded. From the perspective of the adaptive-memory framework, information that not only had to be encoded but also had to be retrieved in a survival context may be especially important to remember. Consistent with this idea, both experiments showed that recognition and source memory were numerically highest when survival processing at encoding was combined with survival-constrained retrieval. This is partially supported by a follow-up analysis that included only those words for which the retrieval query referred to the condition in which the word has initially been encoded, thereby allowing us to isolate the effect of combined survival processing at encoding and retrieval while holding the retrieval-encoding relationship constant. In Experiment 1, words initially judged for survival relevance that had to be retrieved under a survival-constrained retrieval query were descriptively but not significantly better recognized than words initially judged for pleasantness that had to be retrieved under a pleasantness-constrained retrieval query, Δ*G*^2^(1) = 0.45, *p* = 0.504. In Experiment 2, words initially judged for survival relevance that had to be retrieved under a survival-constrained retrieval query were significantly better recognized than words initially judged for moving relevance that had to be retrieved under a moving-constrained retrieval query, Δ*G*^2^(1) = 10.25, *p* = 0.001. Also, source memory was significantly enhanced for words initially judged for survival relevance that had to be retrieved under a survival-constrained retrieval query in comparison to words initially judged for moving relevance that had to be retrieved under a moving-constrained retrieval query, Δ*G*^2^(1) = 63.85, *p* < 0.001.

## Conclusion

In sum, the present findings demonstrate that recognition is enhanced for information that was relevant not only at the time of encoding but also during an intermediate source-constrained retrieval test. Since past experiences often predict future needs, information that has not only been encoded but did already have to be retrieved is likely more relevant for future use than information that did not yet have to be retrieved. The necessity of past retrieval could well be construed as an adaptive marker of the information’s relevance. Even though this interpretation necessarily involves some speculation that goes beyond what is immediately demonstrated by the present results, our results can be understood as supporting the notion of memory’s adaptiveness in this more general sense, emphasizing its dynamic nature in prioritizing information based on its past and potential future relevance.

## Data Availability

The experiments were not preregistered. The data are available at https://osf.io/tf4su/
